# Cerebral Syphilis

**Published:** 1895-01

**Authors:** R. H. A. Boyd

**Affiliations:** San Antonio, Texas


### TEXAS MEDICAL JOURNAL.

ESTABLISHED JULY, 1885.

PUBLISHED MONTHLY.—SUBSCRIPTION $2.00 A YEAR.

VOL. X. AUSTIN, JANUARY, 1895. NO. 7.

### Original Contributions.

For Texas Medical Journal.

### CEREBRAL SYPHILIS.

BY R. H. A. BOYD, A. M., M. D., SAN ANTONIO, TEXAS.

I WILL ask your attention first to a history of a case of cerebral syphilis somewhat in detail, and will then present other histories to illustrate certain phases of the disease.

In September of 1888, I was consulted by a widow, aged fifty, suffering with headache, which she affirmed she had had for five or six years, that they were getting worse, and that nothing had helped her. Upon questioning, she said they were worse at night, and during the day there was a general sore feeling all over the head. She was not able to sleep until near morning. She was easily tired, very fretful, becoming more and more disagreeable to those around her. Her whole moral character was indeed changed. She had two grown children, no miscarriages; had a posterior synechia, the result of an old iritis in the right eye. Scars were seen upon her body; the frontal, sternal, tibial bones were sore and tender on pressure.

There were pains in bones at night. After some jogging of the memory, she said she had been treated ten years previously for blood poisoning, but had not had a thorough course of mercury.

She was put on iodide of potassium, grs. xxx t. i. d., and told to report in a few days.

About a month afterward, I was sent for, and found the patient as follows: Double vision, numbness in arms, limbs and mouth, a partial left hemiplegia, great nausea, and a tendency to stupor.

The family were informed of the necessity of the patient taking the medicine as ordered.

The iodide was increased to a ʒi t. i. d. Special care was taken to see that she was properly nourished. Inside of two weeks the improvement was marked, and the iodide increased to grs. xc t. i. d.

The patient remarked that was the best stomach medicine she had ever taken!

This treatment was kept up for nearly two years. On several occasions the patient stopped the medicine, and there was a relapse. On one occasion, I found her talking incoherently, face congested, tense feeling in the head, pulse full and slow, evidence of brain congestion.

She was put on elaterium and potassium bromide. In a day or so the iodides were resumed, and she was told emphatically if she wanted to live and get well she had to take the medicine. To-day there is no evidence of paralysis, and she is as well as she ever had been.

CASE 2. In the summer of 1885, a policeman brought me a man, aged fifty-five, in coma, with no history of injury, but was asleep on the street. He was not drunk, nor was he suffering from apoplexy, opium poisoning, Bright's disease, or diabetes. I found I could arouse him readily, and he was able slowly to answer some questions. After a somewhat critical examination, I came to the conclusion that he was suffering from brain tumor, which, in all probability, was syphilitic. There was an old scar on his penis, and some tenderness over the long bones. Increasing doses of iodide of potassium were given, and in about two weeks I was able to confirm my diagnosis with the complete history of the case. He was treated for about six weeks, and was then able to be up and around. I then lost track of the patient.

CASE 3. In the spring of 1892, a grass-widow, aged thirty-five, consulted me for double vision. All at once she said she saw double. There was no history of headache, insomnia, or numbness, nor pain in bones. She denied a specific history. There was no history of rheumatism, nor tuberculosis.

I was at a loss for a moment to explain the symptom. The lady was intelligent, had one child, was in good social position.

Tentatively, I put her on iodide of potassium, grs. xx t. i. d., for a week. I told her to rest all she could, and avoid all excitement and mental worry. At the end of the week, there was no improvement, neither were there signs of iodism.

I determined to push the dose until the point of tolerance, and found that a ʒi t. i. d. was all she could stand, and the symptoms began to ameliorate.

I questioned her again more closely, and got a history of syphilis. She said she was only treated for about six months. Judging from what she said, she had the characteristic secondaries.

I am firmly convinced that relapses in nervous symptoms are very frequent, much more so than we are led to believe from the books.

It is a result of either an improper treatment of the early stages, or it is, as Fournier says, seen in cases where the mucous and cutaneous symptoms are slight.

The cases that have been cited will serve as types of the disease, and will serve for a text of what follows:

Nervous syphilis occurs usually in the secondary, or tertiary stage. It may occur in the primary.

I know of a case where the cerebral symptoms appeared three weeks after the initial sore.

We can not exclude cerebral syphilis if there is a lack of clear history, or a lack of the usual cutaneous manifestations. Wood used to teach that any case of obscure nervous disease, if the person had run any risk, no matter whom, should be given the therapeutic test, potassium iodide, grs. xx t. i. d.

There is no structure that syphilis will not assail; the brain substance, the connective tissue, the bones, the membranes of the brain, the arteries and capillaries, fall easy prey. Hence, we have a great variety of symptoms.

If I go somewhat carefully into the pathology of the disease, I hope I will be excused, on account of its importance.

In the blood vessels we have an endarteritis due to the pouring out of round cells from the vasa vasorum. In consequence of which we have a narrowing, or occlusion of the lumen of the vessels, due to the thickening inward of the fenestrated membrane and the intima.

Thus we have a roughened or altered intima ready to act as a nidus for a thrombus, which in turn may produce an embolus. The muscular coat may waste, and form aneurisms.

Around the blood vessels there is a proliferation of cells of connective tissue. These cells are peculiar in that they undergo a calcareous or caseous degeneration.

The bones of the skull may be thickened. The membranes at the base are very much affected, and often adherent to the brain.

The pia looks like wetted blotting paper, in the region of the pons, of the temporo-sphenoidal, and of the posterior parts of the frontal lobes. Granulations will often be found amounting in size to small tumors.

The cranial bones at the base will be found infiltrated and surrounded by these diseased membranes.

As yet, the question of syphilis being due to Lustgarten's bacillus lacks the confirmation of the culture test.

At the present time in the symptomatology of nervous diseases, it is fashionable to show up a train of symptoms. We have a tripod on which rests melancholia. There are three cardinal symptoms of exophthalmic goitre. Nor is cerebral syphilis far behind.

In our therapeutics, too, we have the trinity pill. It will be my endeavor to show what is in the trinity business of this disease.

One word of caution, however; don't expect to find all the symptoms in every case. There may be one or two symptoms left out, their place being supplied by others. Still, the symptoms that are usually given are of great importance, and the grouping of these lead us easily to diagnose our cases. Usually, they are prodromata, such as tired feeling, mental apathy, a changed disposition.

In a person of syphilitic history, these prodromata should warn us of the coming storm. The onset of the storm may be sudden, as in my second case.

There is one symptom which is most constant—headache. It may be a slight ache, or it may be so severe as to cause delirium. The patient will often hold his head between his hands, and groan with pain. These headaches appear toward night. They may appear in the morning. They affect any part of the head. If we meet a headache which is excessively persistent, apparently causeless, with marked nocturnal exacerbations, it is, in all probability, syphilitic.

The second marked symptom is insomnia. This is only seen in the early stage. This symptom, and the headache, disappear on the appearance of the third symptom—convulsions, or paralysis. Thus our triad is formed. Those who insist on this triad say that the memory of the patient is defective, or the symptom so slight as to escape attention. Be this as it may, the grouping of these symptoms should not be wrongly interpreted. In the latter stage, there is a peculiar somnolence.

I have seen it in two forms. First, where it is persistent, as in case two. The patient lies in bed all day, indifferent to everything, can be easily aroused, and answers questions slowly. The second class, the patient goes to sleep at his task, wakes up, and sleeps again. This somnolent condition may last for weeks. It is not necessarily of evil omen. It denotes pressure. Of course, if this is kept up too long, there is a likelihood of brain softening.

The paralysis is usually a hemiplegia, a monoplegia, or a paralysis of one or more cranial nerves. The hemiplegia may be due to a lesion of the cortex, the internal capsule, or the basil ganglia.

The monoplegia is due to a lesion of the motor area of the cortex. This monoplegia may become a hemiplegia. The optic, the olfactory and ocular motor nerves, are the ones usually affected. Rarely are the fourth, fifth, sixth, seventh or eighth nerves involved.

The third nerve is the one most usually affected. The growths of the base are either syphilitic or tubercular. Hence, as a rule, a squint, a ptosis, dilated pupil, or any paralytic eye symptom in the adult, is syphilitic. As a rule, syphilic paralyses are incomplete. This, as it can readily be seen, has a great bearing on prognosis.

A complete hemiplagia denotes either a hemorrhage or a thrombus. The specific palsies are fugitive; they are extensive and incomplete. They are not due, as a rule, to clots, but to congestions in the region of gummas. Among the motor palsies must be mentioned also aphasia, and it has the same characteristic as other motor palsies. Aphasia and left hemiplegia are almost diagnostic of cerebral syphilis, as illustrated by my first case.

Epilepsy is very common after cerebral syphilis. Fournier says that epilepsy occurring after thirty years of age is never essential epilepsy; excluding uremia and alcohol, it is, in nine cases out of ten, specific.

We may have either the petit, or grand mal. The aura is rarely present; when it is seen, it always assumes the same type.

I believe that most cases conform to the Jacksonian type, rather than to the essential.

Consciousness, as a rule, is not lost. Paresis and hallucination are the types of insanity that follow cerebral syphilis.

The diagnosis of cerebral syphilis can be easily made if we have a correct history, but as it has been said before, the diagnosis must often be made without the history. You can readily conceive how a person may be entirely ignorant of the fact of inoculation. Women often know nothing about it. Physicians are known to have been inoculated in various ways. Then, too, people often forget about their early history; they have no desire to call up former indiscretions.

To be brief, a persistent nocturnal headache and insomnia should arouse us to heroic treatment, for we know that a convulsion or a paralysis is staring the individual in the face.

Convulsions after the thirtieth year, not preceded by infantile convulsions nor due to diabetes, traumatism, or to pregnancy or megrims, are syphilitic.

Hemiplegia under forty, is syphilitic.

Symptoms indicative of lesion at the base of the brain in the adult, are syphilitic.

Comatose conditions extending over days, or weeks, not due to traumatism, meningitis, diabetes, nephritis, or typhoid, is syphilitic.

The prognosis, even in seemingly grave cases, is, as a rule, favorable, but it is well for the physician to be on his guard. The lesion may be destructive, as well as nondestructive. Under vigorous treatment, the most desperate cases will get well.

To treat these cases successfully, the physician must have the courage to carry out his convictions. It is no use to give grs. x of iodide of potassium, but the dose must be such that the symptoms either yield or the patient cannot tolerate the drug.

I have never had to give as large doses as some men have, still I would not hesitate to do so if the case demanded it. I have never had to increase the iodide over ℥ij t. i. d. Some have given as much as gr. 800 in the twenty-four hours.

Mercury is rather an uncertain drug in this disease. I have seen cases go halting on the mercury, pick up, and make a rapid recovery under the iodide.

The worst case of salivation I ever saw was in one of these cases. Improvement was noticed very soon under the iodides.

The iodides should be given in a saturated solution, gtt. xx to xxx t. i. d. The dose increased by gtt. iij to v each day, until the point of tolerance is reached, or the symptoms yield. If iodism occur before the symptoms yield, two paths are before us: Either increase the dose by one-third, or keep on increasing daily as before. Strange to say, the iodism under these large doses will disappear, and the large doses are well borne.

But should these large doses increase the iodism, decrease the dose one-half when iodism first appears. Continue then this dose until the iodism entirely disappears, and then commence to increase as before.

The iodides, in some cases, are not well borne, their prognosis is bad.

Small doses, grs. v to x, often affect the heart in some persons. They should be given after meals, largely diluted in ice water or Vichy. The diet, the regimen and hygiene must also be looked after.

Good food, rest from care and worry, are *sine qua non*. The care we bestow on these people often adds as much to recovery as medicine. The one without the other will not cure. I am led to think that relapses are excessively common. We can never know whether a gummatous growth is entirely absorbed. In-ammations around these gummas is the rule.

I have made it a rule to treat these cases actively for two years, and ever after that to advise a course of treatment of six weeks every six months. I instruct these people especially that they had brain syphilis, that they are liable to relapses, and that as time goes on the outbreaks of the disease become more obscure, and if a physician is called who knows nothing of the patient's antecedents, he is apt to blunder. I have seen more than one illustration of this.

